# Demographic Characteristics and Clinical Outcomes of Asian American and Pacific Islander Patients With Primary Intracerebral Hemorrhage 

**DOI:** 10.1001/jamanetworkopen.2021.38786

**Published:** 2021-12-14

**Authors:** Abdulaziz T. Bako, Alan P. Pan, Thomas Potter, Jennifer R. Meeks, Miguel Caínzos-Achirica, Daniel Woo, Farhaan S. Vahidy

**Affiliations:** 1 Center for Outcomes Research, Houston Methodist, Houston, Texas; 2 Division of Prevention and Wellness, Department of Cardiology, DeBakey Heart and Vascular Center, Houston Methodist, Houston, Texas; 3 Department of Neurology and Rehabilitation Medicine, University of Cincinnati, Cincinnati, Ohio

## Abstract

This cross-sectional study analyzes the 15-year demographic, incidence, hospitalization, and case fatality data of Asian American and Pacific Islander adults with intracerebral hemorrhage.

## Introduction

Global data indicate a disproportionate intracerebral hemorrhage (ICH) burden in Asian countries^1^; however, ICH is not well characterized among Asian American and Pacific Islander individuals in the US. This study aimed to provide nationwide estimates of the demographic characteristics and clinical outcomes data of Asian American and Pacific Islander individuals with ICH and to compare them with estimates for Hispanic, non-Hispanic Black, and non-Hispanic White patients.

## Methods

Houston Methodist Institutional Review Board deemed this study exempt from review and informed consent requirement because it used deidentified and publicly available database. We followed the Strengthening the Reporting of Observational Studies in Epidemiology (STROBE) reporting guideline.^2^

We conducted a cross-sectional analysis of adults with primary ICH, which was identified using the validated^3^ *International Classification of Diseases, Ninth Revision,* and *International Statistical Classification of Diseases and Related Health Problems, Tenth Revision* diagnostic codes, using the publicly available National Inpatient Sample database^4^ (under a data use agreement; eMethods in the Supplement) for the 15-year period of January 1, 2004, to December 31, 2018. All analyses used survey weights to provide nationally representative estimates. We calculated mean annual ICH incidence rates (with 95% CIs) overall and for specific racial and ethnic groups using the US Census Bureau population counts. Race and ethnicity were self-reported in the US Census, and the racial and ethnic categories were specified by the US Census Bureau.

In addition, we conducted logistic regression to derive the odds of ICH (vs non-ICH stroke) across racial and ethnic subgroups. We fit a multivariable logistic regression model to identify the association between race and ethnicity and case fatality, and we used a generalized linear model (γ family) to assess the differences in length of stay and hospitalization cost by race and ethnicity. Models were adjusted for demographic, hospital, comorbidity, and treatment characteristics. Estimates were reported as odds ratios (ORs) and mean ratios.

The eMethods in the Supplement provide methodological details. All statistical tests were 2-tailed, and the level of significance was set at α = 0.05. Data analyses were conducted using Stata, version 16 (StataCorp LLC), between May and July 2021.

## Results

The 803 230 total hospitalizations for ICH in the 15-year National Inpatient Sample included 40 582 Asian American and Pacific Islander (5.1%), 80 756 Hispanic (10.1%), 142 240 non-Hispanic Black (17.7%), and 539 471 non-Hispanic White (67.1%) patients with an overall mean (SD) age of 68.9 (13.6) years. Of these patients, 407 090 were men (50.7%), 396 075 were women (49.3%), and they were predominantly insured by Medicare (n = 491 518 [61.2%]). Approximately 60.7% (n = 24 630) of ICH hospitalizations among Asian American and Pacific Islander patients occurred in the Pacific division of the US Census (Alaska, California, Hawaii, Oregon, and Washington).

**Figure 1.**
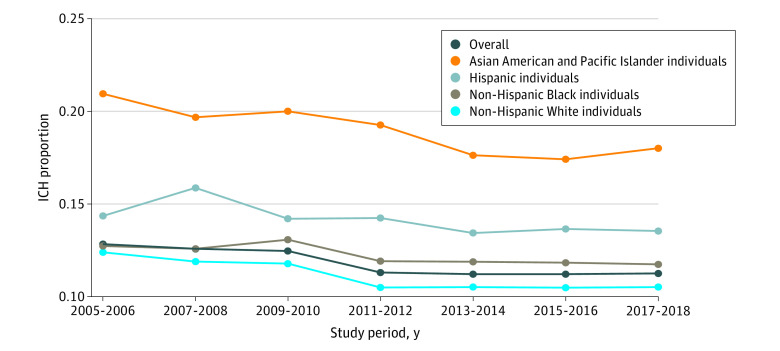
Differences in Proportion of Intracerebral Hemorrhage (ICH) Among All Stroke Subtypes by Race and EthnicityFrom 2005 to 2018

Overall, the mean annual ICH incidence rate per 100 000 individuals was 23.15 (95% CI, 23.10-23.20). By race and ethnicity, the incidence rate per 100 000 was 22.18 (95% CI, 21.97-22.40) for Asian American and Pacific Islander, 15.77 (95% CI, 15.66-15.88) for Hispanic, 34.33 (95% CI, 34.15-34.51) for non-Hispanic Black, and 22.86 (95% CI, 22.80-22.92) for non-Hispanic White patients. The proportion of ICH among all stroke subtypes was highest for Asian American and Pacific Islander patients at 18.9% compared with 14.1% (OR, 1.42; 95% CI, 1.37-1.48) for Hispanic, 12.2% (OR, 1.68; 95% CI, 1.62-1.75) for non-Hispanic Black, and 11.1% (OR, 1.88; 95% CI, 1.82-1.94) for non-Hispanic White patients (Figure 1).

Compared with non-Hispanic White patients, Asian American and Pacific Islander patients experienced ICH at a younger age (66.3 vs 72.0 years), and a higher proportion of these patients were privately insured (26.8% vs 19.2%) and resided in large metropolitan areas (76.7% vs 48.6%) or in zip codes with the highest median household income (40.7% vs 25.1%). Furthermore, Asian American and Pacific Islander vs non-Hispanic White patients had a higher burden of hypertension (86.5% vs 79.2%), hyperlipidemia (37.4% vs 35.1%), ischemic stroke (10.6% vs 8.8%), and diabetes (23.4% vs 18.5%). However, Asian American and Pacific Islander vs non-Hispanic White patients had a lower proportion of atrial fibrillation (16.3% vs 23.6%) and previous or current anticoagulant use (7.8% vs 12.0%). A larger proportion of Asian American and Pacific Islander patients compared with their non-Hispanic White counterparts had extreme loss of function on an illness severity scale (31.8% vs 25.4%) and were intensively managed with invasive ventilation (9.3% vs 6.7%), tracheostomy (5.4% vs 2.9%), extraventricular drain (9.6% vs 6.0%), and gastric tube placement (10.7% vs 6.8%).

**Figure 2.**
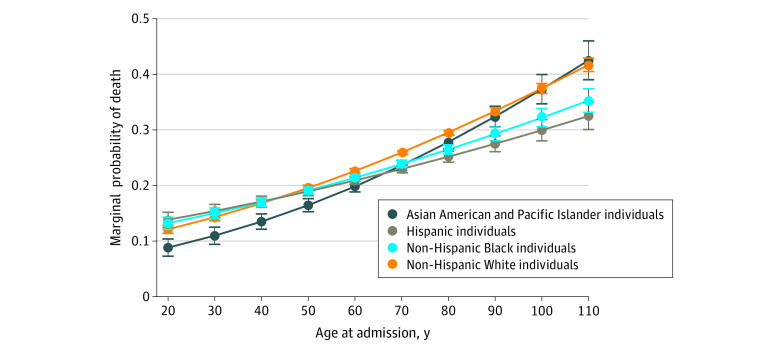
Differences in Likelihood of Intracerebral Hemorrhage Case Fatality Across an Age Spectrum by Race and Ethnicity

A significant age–race and ethnicity interaction for ICH case fatality was observed, whereby the age-associated increase in ICH case fatality was significantly higher among Asian American and Pacific Islander individuals compared with those from other race and ethnicity subgroups (Figure 2). Compared with non-Hispanic White patients, Asian American and Pacific Islander patients had a longer length of stay (mean ratio, 1.09; 95% CI, 1.06-1.11) and a higher hospitalization cost (mean ratio, 1.02; 95% CI, 1.01-1.04).

## Discussion

The Asian American and Pacific Islander group is a rapidly growing but understudied population in the US.^5^ This study found that these individuals had a comparable incidence rate yet higher odds of ICH (vs non-ICH stroke) compared with those from other racial and ethnic groups. Despite their younger age, Asian American and Pacific Islander patients (vs non-Hispanic White patients) had a higher ICH-related comorbidity burden and ICH severity, which coincided with the longer length of stay, higher hospitalization costs, and greater age-associated ICH case fatality for this group. Despite the limitations of using *International Classification of Diseases*–based case identification and the lack of neuroimaging or physiological data, this study provides contemporary nationwide estimates of ICH burden among Asian American and Pacific Islander patients. These findings warrant further analyses of the unique environmental and genetic risk factors that predispose the Asian American and Pacific Islander population to ICH.

## Notes

Supplement.
eMethods

eReferences
