# Supplementary material for: Demographic Characteristics and Clinical Outcomes of Asian American and Pacific Islander Patients With Primary Intracerebral Hemorrhage
Source: JAMA Netw Open. 2021 Dec 14;4(12):e2138786. doi: 10.1001/jamanetworkopen.2021.38786 (PMC8672230; doi:10.1001/jamanetworkopen.2021.38786)
Supplement: Supplement. — eMethods eReferences [file jamanetwopen-e2138786-s001.pdf]

Supplementary Online Content

Bako AT, Pan AP, Potter T, et al. Demographic characteristics and clinical outcomes of Asian American and Pacific Islander patients with primary intracerebral hemorrhage. *JAMA Netw Open*. 2021;4(12):e2138786. doi:10.1001/jamanetworkopen.2021.38786

eMethods

eReferences

This supplementary material has been provided by the authors to give readers additional information about their work.

© 2021 Bako AT et al. *JAMA Network Open.*

eMethods

Data source: The National Inpatient Sample (NIS) is the largest publicly available all-payer (including the uninsured) inpatient database in the US.[1] For Medicare patients, NIS includes Medicare Advantage patients, a population often missed in Medicare claims analyses, even though it comprises up to 30% of Medicare beneficiaries.[2] The 2018 NIS includes data from 47 states and district of Columbia covering 97% of US population and approximately 96% of community hospital discharges in the US.[2] NIS is deidentified and is provided by the Healthcare Cost and Utilization Project of the Agency for Healthcare Research and Quality under a data use agreement.[1] Census Bureau's public use microdata sample (PUMS) was used to obtain population counts for the overall sample and specific race/ethnicity subgroups, which were used as denominators in incidence rate calculations.

Cohort Identification: Our study cohort include adult (age ≥ 18 years) patients with a primary ICH diagnosis, identified using International Classification of Disease, Ninth Revision (ICD-9) code 431 and Tenth Revision (ICD-10) codes I61 (I61.0 – I61.6 and I61.8 – I61.9). We excluded encounters with concurrent diagnoses of head trauma or arteriovenous malformation and encounters with Native American, "Other" race and missing race variable.

Outcome variables: The outcomes are in-hospital mortality, length of stay (LOS), and hospitalization cost. In-hospital mortality (case fatality) is provided as a pre-coded variable in NIS. LOS was defined as the difference (days) between discharge and admission date. Same-day events were coded as zero. We calculated costs for each encounter by converting hospital charges to costs using HUCP's cost-to-charge ratio files.[3] Costs were adjusted for inflation to 2018 US dollar equivalent using the Chained Consumer Price Index for all urban consumers and medical care services from the US Bureau of Labor Statistics.[4] All variables are consistently provided across the years analyzed.

Statistical Analyses: All analyses were conducted using survey design methods, with appropriate discharge and trend weights utilized to yield nationally representative estimates. For incidence rate calculations, we used survey design variables with appropriate discharge and trend weights to yield nationally representative estimates of the aggregate counts of yearly ICH incidence across race/ethnicity subgroups. Census Bureau's PUMS provided the time-specific population counts used as denominators. We provide race/ethnicity-specific rates of ICH hospitalizations, as proportions and 95% confidence intervals (CI) of total stroke hospitalizations across the 15-year period. Additionally, we fit panel data random effects Poisson regression model with robust standard errors to report the crude incidence rate ratios (IRR) with CI for race/ethnicity differences in ICH incidence. For outcomes assessment, we fit logistic regression models and report adjusted odds ratios (aOR) and CI as a national estimate of the likelihood of mortality among AAPI ICH patients compared to NHW. We also report interaction effect of age with race/ethnicity on likelihood of mortality. The age-race/ethnicity interaction was prespecified because age is one of the strongest predictors of ICH-related mortality[5] and we intended to investigate whether this association varies across race/ethnicity sub-groups. We report adjusted mean ratios (MR) for likelihood of longer LOS and higher hospitalization cost by fitting the

© 2021 Bako AT et al. *JAMA Network Open.*

gamma family of generalized linear model with log link function. Analyses were performed using STATA (version 16).

All multivariable models were adjusted for 1) Demographic variables: age, sex, race/ethnicity, payment/insurance type, year of analysis, patient location, median household income quartile for patient zip code; 2) Hospital characteristics: census division of hospital location, urban-rural / teaching status of hospital; 3) Comorbidities and risk factors: alcohol abuse, deficiency anemias, congestive heart failure, chronic lung disease, combined complicated and uncomplicated hypertension, uncomplicated diabetes, diabetes with complications, paralysis, peripheral vascular disease, peptic ulcer, history of ischemic stroke, history of TIA, hyperlipidemia, past or current history of smoking, long term or current use of anticoagulants, atrial fibrillation; and 4) Intensity of treatment and disease severity metrics: extra ventricular drain placement, hemicraniectomy / hemicraniotomy, ventilator support (invasive), ventilator support (non-invasive), tracheostomy, gastric tube, and the all patient refined diagnosis related groups (APR-DRG) severity of illness score.

© 2021 Bako AT et al. *JAMA Network Open.*

eReferences

1. NIS Database Documentation. Accessed June 27, 2021. https://hcup-us.ahrq.gov/db/nation/nis/nisdbdocumentation.jsp

2. Introduction to the HCUP National Inpatient Sample (NIS) 2018. Published online November 2020. https://www.hcup-us.ahrq.gov/db/nation/nis/NISIntroduction2018.pdf

3. HCUP-US Cost-to-Charge Ratio for Inpatient Files. Accessed June 27, 2021. https://www.hcup-us.ahrq.gov/db/ccr/ip-ccr/ip-ccr.jsp

4. U.S. Bureau of Labor Statistics. CPI for All Urban Consumers (CPI-U). Accessed June 20, 2021. https://data.bls.gov/cgi-bin/surveymost?cu

5. van Asch CJ, Luitse MJ, Rinkel GJ, van der Tweel I, Algra A, Klijn CJ. Incidence, case fatality, and functional outcome of intracerebral haemorrhage over time, according to age, sex, and ethnic origin: a systematic review and meta-analysis. *Lancet Neurol*. 2010;9(2):167-176. doi:10.1016/S1474-4422(09)70340-0

© 2021 Bako AT et al. *JAMA Network Open.*
